# Smoking, alcohol use disorder and tuberculosis treatment outcomes: A dual co-morbidity burden that cannot be ignored

**DOI:** 10.1371/journal.pone.0220507

**Published:** 2019-07-31

**Authors:** Beena Elizabeth Thomas, Kannan Thiruvengadam, Rani S., Dileep Kadam, Senthanro Ovung, Shrutha Sivakumar, Shri Vijay Bala Yogendra Shivakumar, Mandar Paradkar, Nikhil Gupte, Nishi Suryavanshi, C. K. Dolla, Akshay N. Gupte, Rewa Kohli, Neeta Pradhan, Gomathi Narayan Sivaramakrishnan, Sanjay Gaikwad, Anju Kagal, Kavitha Dhanasekaran, Andrea Deluca, Jonathan E. Golub, Vidya Mave, Padmapriyadarshini Chandrasekaran, Amita Gupta, for the CTRIUMPH-RePORT India Study

**Affiliations:** 1 National Institute for Research in Tuberculosis, ICMR, Chennai, Tamil Nadu, India; 2 Byramjee Jeejeebhoy Government Medical College, Pune, Maharashtra, India; 3 Johns Hopkins University–India Office, Pune, Maharashtra, India; 4 Byramjee Jeejeebhoy Government Medical College–Johns Hopkins University Clinical Research Site, Pune, Maharashtra, India; 5 Johns Hopkins School of Medicine, Baltimore, Maryland, United States of America; 6 Johns Hopkins Bloomberg School of Public Health, Baltimore, Maryland, United States of America; University of Calfornia San Francisco, UNITED STATES

## Abstract

**Background:**

More than 20% of tuberculosis (TB) disease worldwide may be attributable to smoking and alcohol abuse. India is the second largest consumer of tobacco products, a major consumer of alcohol particularly among males, and has the highest burden of TB globally. The impact of increasing tobacco dose, relevance of alcohol misuse and past versus current or never smoking status on TB treatment outcomes remain inadequately defined.

**Methods:**

We conducted a multi-centric prospective cohort study of newly diagnosed adult pulmonary TB patients initiated on TB treatment and followed for a minimum of 6 months to assess the impact of smoking status with or without alcohol abuse on treatment outcomes. Smokers were defined as never smokers, past smokers or current smokers. Alcohol Use Disorder Identification Test (AUDIT) scores were used to assess alcohol misuse. The association between smoking status and treatment outcomes was assessed in univariate and multivariate random effects poisson regression models.

**Results:**

Of 455 enrolled, 129 (28%) had a history of smoking with 94 (20%) current smokers and 35 (8%) past smokers. Unfavourable treatment outcomes were significantly higher among past and current smokers as compared to never smokers. Specifically, the risk of treatment failure was significantly higher among past smokers (aIRR = 2.66, 95% CI: 1.41–4.90, p = 0.002), recurrent TB among current smokers (aIRR = 2.94, 95% CI: 1.30–6.67, p = 0.010) and death among both past (2.63, 95% CI: 1.11–6.24, p = 0.028) and current (aIRR = 2.59, 95% CI: 1.29–5.18, p = 0.007) smokers. Furthermore, the combined effect of alcohol misuse and smoking on unfavorable treatment outcomes was significantly higher among past smokers (aIRR: 4.67, 95% CI: 2.17–10.02, p<0.001) and current smokers (aIRR: 3.58, 95% CI: 1.89–6.76, p<0.001).

**Conclusion:**

Past and current smoking along with alcohol misuse have combined effects on increasing the risk of unfavourable TB treatment outcomes. Innovative interventions that can readily address both co-morbidities are urgently needed.

## Introduction

Tuberculosis (TB) is the ninth leading cause of death and the top infectious disease killer worldwide. The WHO Global Tuberculosis Report 2018 has reported that approximately 10 million people fell ill with TB in 2017 and India alone accounted for 27% (2.74 million) of the world’s TB case burden [1,2].

While there is effective treatment for TB, 10% to 30% of people with TB have composite outcomes of treatment failure, recurrence or death [2–6]. There are many important potentially modifiable risk factors associated with these unfavorable TB treatment outcomes, including tobacco smoking and alcohol use. Other risk factors include diabetes mellitus, HIV and low body mass index (BMI) [7,8]. Both tobacco smoking and alcohol use often occur together and use patterns are highly variable. While numerous studies have looked at these factors and have reported on the associations, an important shortcoming is the lack of collective, prospective ascertainment of these numerous risk factors as well as the assessment of impact of dose effect (e.g. past vs. current tobacco and amount of tobacco and alcohol consumed) and combined effects of tobacco smoking and alcohol use, adjusting for confounding effects.

We sought to assess the impact of smoking status (never, past and current) and its combined effect with alcohol use status given both are highly prevalent and likely have important interactions in influencing TB treatment outcomes in India.

## Methodology

This study was conducted as part of the prospective ‘CTRIUMPh’ RePORT India cohort [9]. CTRIUMPh has been enrolling and following adult (≥18 years) pulmonary TB cases at the National Institute for Research in Tuberculosis (NIRT) in Chennai, India and the Byramjee-Jeejeebhoy Government Medical College–Sassoon General Hospitals (BJGMC-SGH) in Pune, India, since August 2014 through academic and operational partnership with the Johns Hopkins University (JHU), Baltimore, USA.

Pulmonary TB cases were diagnosed by the presence of acid-fast bacilli (AFB) on smear microscopy, *Mycobacterium tuberculosis (Mtb)* DNA on Xpert MTB/RIF assay, Mtb growth on liquid or solid culture, or based on clinical judgment in the absence of microbiological confirmation of TB. Cases with drug resistant disease, those with prior TB and pregnant women with TB were excluded. Participants were enrolled within 7 days of TB treatment initiation and prospectively followed for up to 24 months. Chest radiography evaluation at enrolment identified cavitary lung disease.

Standardized semi-structured interview schedules and operating procedures were used to collect socio-demographic, clinical and laboratory data. The Fagerström Test for Nicotine Dependence was used as a standard instrument for assessing the intensity of physical addiction to nicotine [10]. The Alcohol Use Disorder Identification Test (AUDIT), a 10-item screening questionnaire for hazardous and harmful alcohol consumption and alcohol related problems was used to assess alcohol use [11]. The Centre for Epidemiologic Studies Depression Scale (CES-D), a 20 item scale was used to measure symptoms of depression as defined by the American Psychiatric Association Diagnostic and Statistical Manual, fifth edition [12].

### Definitions

#### Tobacco smoke use

Tobacco smoking was defined as follows at study entry: never smokers were those who smoked <100 cigarettes in their lifetime and were not current smokers; past smokers were those who smoked ≥ 100 cigarettes in their lifetime and were not currently smoking; and current smokers were those who smoked ≥100 cigarettes in their lifetime and reported current smoking. Pack years was calculated by multiplying the number of years smoked with the average number of packs (20 smoked tobacco products/pack) per day.

#### Alcohol use

AUDIT scoring system was used to define levels of alcohol use, those with the minimum score being 8. Total scores of 8–15, 16, 17–19, and ≥20 indicate alcohol dependence, harmful use, alcohol abuse and hazardous use, respectively [13].

#### Treatment outcomes

Composite outcome that included death (all causes of mortality), treatment failure (microbiological evidence of TB during last 2 months of treatment (month 5 or 6) by culture or AFB microscopy or clinical judgement if microbiological evidence was unavailable) and recurrence (microbiological evidence of TB after the successful completion of treatment by culture or AFB microscopy or clinical judgement, if microbiological evidence unavailable).

Favourable outcomes were defined as cured with evidence of consecutive negative *M*.*tb* during the last 2 months of TB treatment and treatment completed as absence of bacteriological evidence and/or absence of symptoms suggestive of TB at completion of TB treatment.

### Statistical approaches

The analysis included comparison of baseline characteristics against the smoking profile using Mann Whitney, Kruskal-Wallis with post-hoc test. We also analysed the association between baseline characteristics with TB treatment outcome using Fisher’s Exact test. Person-time was calculated in years from the time of initiation into TB treatment to the occurrence of the unfavourable TB treatment outcome or until the last observed time of visit. Furthermore, the incidence rate of outcome was calculated over the person time period.

Univariate and multivariate Poisson regression with person-time as offset was used to identify the risk for unfavourable TB treatment outcome due to smoking. For the multivariate analysis, variables known to be associated with composite and individual treatment outcomes were identified through a review of published literature. In addition, an exploratory data analysis was also done to identify the effect modifiers of the association between TB treatment outcome and smoking status using Breslow-Day test of homogeneity. From this analysis, alcohol consumption was considered as an effect modifier, which remained in the multivariate model as an interaction term with smoking status.

Sensitivity analysis was performed to assess the influence of gender on the association between smoking status and TB treatment outcome. The dose response analysis was done to assess the relationship between intensity, frequency and duration of smoking with TB treatment outcomes. A cigarette pack year was taken as a proxy for smoking intensity. Statistical significance was determined at p<0.05. The statistical analysis was done using Stata V.15.0 (StataCorp, USA).

Written informed consent from the study participants was obtained. Ethics approval for the project was obtained from Institutional Review Boards of NIRT, BJGMC–SGH and Johns Hopkins School of Medicine.

## Results

### General profile of study cohort

Of 455 participants enrolled, 295 (65%) were male. The median age was 38 years (IQR: 27–49), 78 (17%) had no formal education, 304 (67%) were employed, 261 (57.4%) had a BMI of <18.5 and 45 (10%) had an AUDIT ≥ 8. A total of 326 (72%) were never smokers, 35 (8%) were past smokers and 94 (20%) were current smokers. Based on the Fagerström scale for nicotine dependence, 56 (60%) of current smokers were classified as having “low-to-moderate” dependence and 21 (22%) had “moderate-to-high” dependence. Microbiology assessment at study entry identified 305 (67%) smear positive for AFB and 378 (83%) culture positive for *Mtb* (Table 1).

**Table 1:** Comparison of profile between smoker vs. never smokers.

Factors	Never n = 326	Past n = 35	Current n = 94	P-value
Age (in years)^*^	34 (25–45)	45 (30–52)	48.5 (39–54)	<0.001
**Gender**				
Female	159 (49%)	0 (0%)	1 (1%)	<0.001
Male	167 (51%)	35 (100%)	93 (99%)	
**Alcohol use**				
Audit < 8	312 (96%)	24 (69%)	74 (79%)	<0.001
Audit ≥ 8	14 (4%)	11 (31%)	20 (21%)	
**BMI (kg/m**^**2**^**)**				
< 16.0	74 (23%)	12 (34%)	34 (36%)	0.053
16.0–18.5	102 (31%)	12 (34%)	27 (29%)	
≥ 18.5	150 (46%)	11 (31%)	33 (35%)	
**Education**				
Literate	276 (85%)	27 (77%)	74 (79%)	0.227
Illiterate	50 (15%)	8 (23%)	20 (21%)	
**Occupation**				
Non-Working	104 (32%)	17 (49%)	30 (32%)	0.142
Working	222 (68%)	18 (51%)	64 (68%)	
**Family Income**				
<15000	259 (79%)	29 (83%)	76 (81%)	0.922
>15000	67 (21%)	6 (17%)	18 (19%)	
**Area**				
Rural	74 (23%)	16 (46%)	41 (44%)	<0.001
Urban	252 (77%)	19 (54%)	53 (56%)	
**Diabetes**				
Absence	240 (74%)	20 (57%)	66 (70%)	0.111
Presence	86 (26%)	15 (43%)	28 (30%)	
**Smoke Age**	NA	18 (15–20)	20 (16–21)	0.110
**Smoke Duration**	NA	26 (13–37)	30 (14–36)	0.489
**Pack Year**	NA	8.6 (1.4–31)	7.4 (2.6–22)	0.481
**Centre for Epidemiologic Studies Depression Scale (CES)**				
Normal	169 (52%)	16 (46%)	54 (57%)	0.452
Depressed	157 (48%)	19 (54%)	40 (43%)	
CES Score	n = 326; 9 (5–14)	n = 35; 10 (3–17)	n = 94; 8 (5–14)	0.814
**Cavity**				
Absence	216 (66%)	15 (43%)	56 (60%)	0.019
Presence	110 (34%)	20 (57%)	38 (40%)	
**HIV**				
Negative	300 (92%)	34 (97%)	91 (97%)	0.212
Positive	26 (8%)	1 (3%)	3 (3%)	
**Smear**				
Negative	121 (37%)	7 (20%)	22 (23%)	0.011
Positive	205 (63%)	28 (80%)	72 (77%)	
**Culture**				
Negative	66 (20%)	2 (6%)	9 (10%)	0.009
Positive	260 (80%)	33 (94%)	85 (90%)	

Values were shown in n (%) and median (Inter-Quartile Range)

Fisher's Exact test was used to compare the categorical information and K-Wallis followed by Dunn post-test that was used to compare age and CES score at 5% level of significance

*"Never" is different from the other two groups

### Comparison of profile of current smokers, past smokers and never smokers

The profile of smokers both past and current were similar and significantly different to never smokers. Specifically, the median age of current smokers, past smokers and never smokers was 48.5, 45 and 34 years respectively (p<0.001), living in urban areas (56% vs. 54% vs. 77%, p<0.001), BMI <16.0 (36% vs. 34% vs. 23%, p = 0.053), cavitary TB (40% vs. 57% vs. 34%, p = 0.019), smear positivity (77% vs. 80% vs. 63%, p = 0.011) and Mtb culture positivity (90% vs. 94% vs. 80%, p = 0.009). There was also a significant difference in AUDIT scores ≥8 by smoking status (current 21% vs past 31% vs never smokers 4%, p<0.001). Smoking and alcohol use among women was negligible; except for one woman, smoking was reported only among men (Table 1).

### Smoking history

The median age of initiation of smoking among current smokers was 20 years (IQR: 16–21) with a median average smoking duration of 30 years (IQR: 14–36). Similarly, the age of initiation of smoking among past smokers was 18 years (IQR: 15–20) with an average smoking duration of 26 years (IQR: 13–37). The median number of packs smoked in a year by current smokers was 7.4 (IQR: 2.6–22.0) and by past smokers was 8.6 (IQR: 1.4–31.0) (Table 1). Among smokers, 83 (64%) were cigarette smokers, 79 (61%) smoked bidis and 28 (22%) smoked both (Not tabulated).

### Overall TB treatment outcomes

The median follow-up time was 18 months, which constituted 623 person-years of risk. Overall, 81(18%) participants had composite (failure, recurrence and death) treatment outcomes with an incidence rate of 122 per 1000 person-years, of which 40 (9%) were failure with an incidence rate of 61 per 1000 person-years, 20 (4%) were recurrence with an incidence rate of 30 per 1000 person-years and 21 (5%) were death with an incidence rate of 45 per 1000 person-years (Not tabulated).

### Risk factors associated with composite outcomes (failure, recurrence and death)

**Fig 1:**
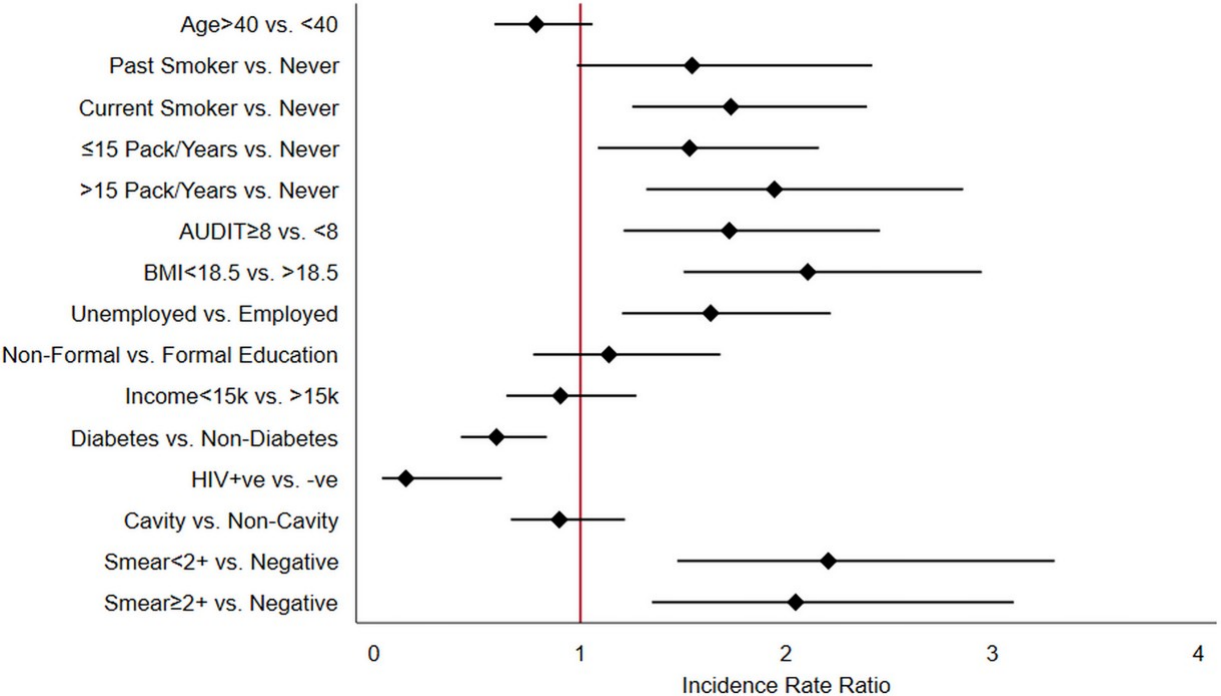
Univariate assessment of risk factors of composite TB treatment outcomes among males.

Females were excluded in further analysis of smoking and alcohol use given only one woman reported these behaviors. Univariate analysis shows that current smoking (IRR = 1.73, 95% CI: 1.25–2.39, p = 0.001), pack years of smoking >15 (IRR = 1.94, 95% CI: 1.32–2.86, p = 0.001), alcohol dependence i.e. AUDIT score ≥8 (IRR = 1.72, 95% CI: 1.21–2.45, p = 0.003), severe underweight i.e. BMI <18.5 (IRR = 2.10, 95% CI: 1.50–2.95, p<0.001), unemployment (IRR = 1.63, 95% CI: 1.20–2.21, p = 0.002) and higher AFB smear grade were risk factors associated with composite treatment outcomes (Fig 1).

### Smoking history and TB treatment outcomes

**Fig 2:**
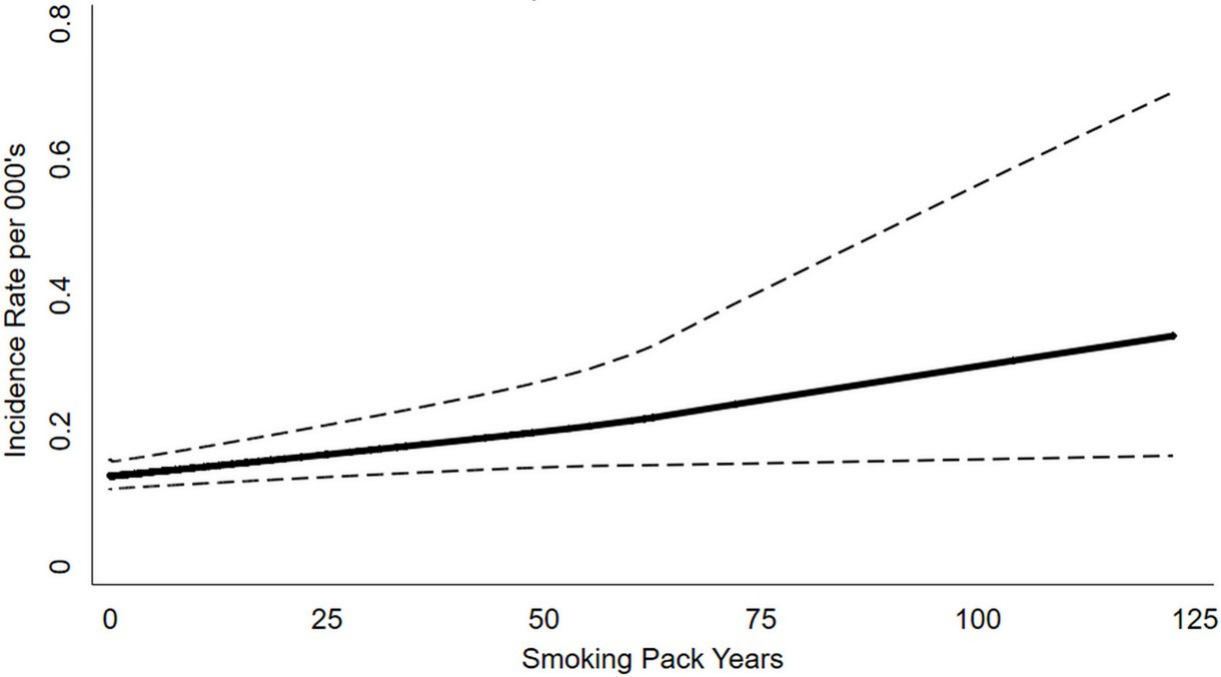
Dose-response relationship between tobacco smoking and the risk of composite TB treatment outcomes.

Multivariable analysis found that being a smoker i.e. past smoker (aIRR = 2.20, 95% CI: 1.27–3.81, p = 0.005) or current smoker (aIRR = 2.03, 95% CI: 1.33–3.08, p = 0.001) had a higher risk for composite TB treatment outcome after adjusting the potential confounders. Assessing individual treatment outcomes, the risk of failure (aIRR = 2.66, 95% CI: 1.41–4.90, p = 0.002) and recurrent TB (aIRR = 2.94, 95% CI: 1.30–6.67, p = 0.010) was significantly higher in past and current smokers respectively, compared to never smokers. The risk of death was higher among both current smokers (aIRR = 2.59, 95% CI: 1.29–5.18, p = 0.007) and past smokers (2.63, 95% CI: 1.11–6.24, p = 0.028) (Table 2). The dose response analysis reflects an increase in the risk of composite outcomes with increase in pack years (Fig 2).

**Table 2:** Risk of composite TB treatment outcomes due to smoking and alcohol among males.

Smoking & Alcohol	Unadjusted		Adjusted^2^	
	IRR (95%CI)	p-value	aIRR (95%CI)	p-value
**Composite**^**1**^				
Never & AUDIT <8	Reference		Reference	
Never & AUDIT ≥8	1.09 (0.48–2.51)	0.834	1.01 (0.36–2.84)	>0.950
Past & AUDIT <8	1.30 (0.72–2.35)	0.377	1.54 (0.76–3.13)	0.227
Past & AUDIT ≥8	1.95 (1.06–3.59)	0.032	4.67 (2.17–10.02)	<0.001
Current & AUDIT <8	1.50 (1.04–2.17)	0.031	1.75 (1.10–2.79)	0.019
Current & AUDIT ≥8	2.62 (1.62–4.21)	<0.001	3.58 (1.89–6.76)	<0.001

IRR = incidence rate ratio; aIRR = adjusted IRR

^1^Composite treatment outcome included failure, recurrence and death

^2^Adjusted for age, BMI, family income, HIV coinfection, diabetes, chest x-ray cavity and smear.

### Combined effect of smoking and alcohol use on TB treatment outcomes

There was a significant change in the magnitude of the association between smoking and TB treatment outcomes when alcohol use was added into the model. Specifically, a synergistic effect of smoking and alcohol on TB treatment outcomes was observed. Compared to those who never smoked nor had alcohol use disorder, past smokers with alcohol dependence (4.67, 95% CI: 2.17–10.02, p<0.001) and current smokers with alcohol dependence (3.58, 95% CI: 1.89–6.76, p<0.001) had the highest observed risk of composite outcomes (Table 2).

## Discussion

Our cohort study has highlighted several key findings on the relationship between smoking status, dose and duration and treatment outcomes; the gender difference in prevalence of smoking and alcohol and the combined impact of smoking and alcohol misuse on composite outcomes. Our study highlights the critical need to address smoking and alcohol misuse with novel interventions given their significant negative sequel on TB treatment outcomes in India, the country with the greatest burden of TB.

Firstly, we observed that overall 18% of our cohort followed up for 18 months had an unfavorable outcome (failure, recurrence and death). More than 25% of TB patients reported history of smoking, either current or past smoking. Our study findings clearly indicate that being a smoker is a significant risk factor for composite treatment outcomes. Several studies have observed the association between smoking and TB treatment outcomes including studies from India, Georgia, Pakistan, Brazil and Armenia [14–17]. What has been less commented on is the dose and duration of smoking and TB treatment outcomes. We specifically assessed the impact of past and current smoking as well as dose response on TB treatment outcomes and observed that past and current smoking as well as cumulative exposure are associated independently with composite treatment outcomes. The risk of failure, recurrence and death was significantly higher among past and current smokers as compared to never smokers. Two cohort studies conducted in Malaysia and Morocco found that smoking increased 2–7 fold the odds of treatment failure [18,19]. This relationship between smoking and TB recurrence as well as smoking and death has also been observed [15,20–24]. A history of past smoking has prognostic importance for failure and dealth, and current smoking assessments would help provide timely intervention, which is important to prevent recurrence and failure. However, current smokers are the only focus of attention for any TB treatment intervention with the advice often being to quit smoking [25–28]. Our study findings also caution us not to confine to only an assessment of current smoking but to also give equal importance to eliciting the past history of smoking.

A dose response relationship in our study shows that an increase in the duration of smoking among participants with a history of smoking does not increase the risk of composite outcomes. This is contrary to another study in Congo, which reports that smoking was independently associated with TB treatment outcomes among those who reported smoking ≥10 years [29].

Secondly, only one female reported smoking, illustrating a clear sex-specific difference in risk factors contributing to unfavorable TB treatment outcomes. The GATS survey of TB tobacco smoking in India also found fewer women reported smoking compared to men, i.e., 19% of men and 2% of women reported smoking [30,31]. While the overall smoking prevalence among men and women has been growing in India, studies have reported that female smoking is growing at a faster rate than that of males but smoking prevalence among women is seldom reported [24,32]. This could be mainly because of the stigma attached to smoking among women considering the cultural norms [33,34]. This gap needs to be addressed and cannot be ignored while eliciting smoking history among TB patients.

Finally, we specifically assessed combined effect of past or current smoking and alcohol misuse on TB treatment outcomes. It was seen that males with past or current tobacco use along with alcohol misuse have the highest risk of unfavorable TB treament outcomes. This clearly highlights the importance of addressing both disorders together and initiating effective interventions for reducing tobacco and alcohol use during TB treatment. Prior studies have shown that alcohol consumption is associated with an increased risk of TB and is a major contributor to the TB burden of disease [35–40]. Two earlier studies have also reported that smoking and drinking habits tend to be linked together and are individually associated with unfavorable treatment outcomes [38,39]. However, it is suprising that there are relatively few data on the combined effect since these disorders are usually inextricably linked.

We also observed that being severely underweight was significantly associated with composite treatment outcomes. It was found that there was a significant difference in the profile of BMI between smokers and never smokers, which has also been reported in other studies [41–44]. Hence, low BMI along with smoking is an important history that health care providers need to be aware of in understanding treatment outcomes.

## Limitations

Our study has some limitaitons. Since we have used self-reported smoking status and self reported AUDIT questionnaire and have not measured nicotine and alcohol or PEth levels, it is possible that we underestimated the smoking and alcohol prevalence and amount and their relationship with TB treatment outcomes. We also did not include smokeless tobacco use such as chewing tobacco which is often more prevalent among women.We may also have unmeasured confounders that contributed to unfavorable treatment outcomes. We did not measure Mtb strain types or host genetics that have been associated with TB outcomes. However, we systematically assessed multiple comorbidities and potential confounders in our prospective cohort; data which are often not readily available.

## Conclusion

Tobacco smoking is an important risk factor that needs to be urgently addressed to decrease adverse composite TB treatment outcomes. We further highlight the importance of eliciting a history of both past and current smoking, given the impact on mortality. We show that current smoking is associated with TB recurrence and the synergistic negative sequalae of combined smoking and alcohol misuse. Health care providers need to be equipped to use standardised measurements such as Ferguson and AUDIT to ascertain smoking and alcohol misuse for all TB patients. Lastly, a holistic approach to smoking cessation that addresses multimorbidity, particularly alcohol misuse, and undernutrion is crtitical for improving
