# Correction: Smoking, alcohol use disorder and tuberculosis treatment outcomes: A dual co-morbidity burden that cannot be ignored

**DOI:** 10.1371/journal.pone.0224914

**Published:** 2019-11-01

**Authors:** Beena Elizabeth Thomas, Kannan Thiruvengadam, Rani S., Dileep Kadam, Senthanro Ovung, Shrutha Sivakumar, Shri Vijay Bala Yogendra Shivakumar, Mandar Paradkar, Nikhil Gupte, Nishi Suryavanshi, C. K. Dolla, Akshay N. Gupte, Rewa Kohli, Neeta Pradhan, Gomathi Narayan Sivaramakrishnan, Sanjay Gaikwad, Anju Kagal, Kavitha Dhanasekaran, Andrea Deluca, Jonathan E. Golub, Vidya Mave, Padmapriyadarshini Chandrasekaran, Amita Gupta, for the CTRIUMPH-RePORT India Study

In the Methodology section, there is an error in the third sentence of the third paragraph. The correct sentence is: The Alcohol Use Disorder Identification Test (AUDIT-C), a 3-item screening questionnaire for hazardous and harmful alcohol consumption was used to assess alcohol use [11].

In the Alcohol Use subsection of the Definitions subsection of the Methodology section, there is an error in the sentence. The correct sentence is: AUDIT-C was used to define alcohol misuse, those with a score of 8 or higher were defined as heavy alcohol misuse [13].

The questionnaire name AUDIT appears incorrectly throughout the article. The correct name is AUDIT-C.

There is an error in Reference 11. The correct reference is: Bush K, Kivlahan DR, McDonell MB, Fihn SD, Bradley KA. The AUDIT alcohol consumption questions (AUDIT-C): an effective brief screening test for problem drinking. Ambulatory Care Quality Improvement Project (ACQUIP). Alcohol Use Disorders Identification Test. Arch Intern Med. 1998 Sep 14; 158(16):1789–95. PubMed PMID: 9738608.

There is an error in Reference 13. The correct reference is: Nayak, MB; Bond, JC; Cherpitel, C; Patel, V; Greenfield, TK; (2009) Detecting Alcohol-Related Problems in Developing Countries: A Comparison of 2 Screening Measures in India. Alcoholism, clinical and experimental research. ISSN 0145-6008 DOI: https://doi.org/10.1111/j.1530-0277.2009.01045.x
